# Applying Implicit Association Test Techniques and Facial Expression Analyses in the Comparative Evaluation of Website User Experience

**DOI:** 10.3389/fpsyg.2021.674159

**Published:** 2021-10-12

**Authors:** Maurizio Mauri, Gaia Rancati, Andrea Gaggioli, Giuseppe Riva

**Affiliations:** ^1^Department of Psychology, Catholic University of Milan, Milan, Italy; ^2^Department of User Experience and Marketing Research, SR LABS, Milan, Italy; ^3^Department of Business and Economics, Allegheny College, Meadville, PA, United States; ^4^Applied Technology for Neuro-Psychology Lab, I.R.C.C.S. Istituto Auxologico Italiano, Milan, Italy

**Keywords:** facial expression, emotions, user experience (UX), brand association, online experiment

## Abstract

This research project has the goal to verify whether the application of neuromarketing techniques, such as implicit association test (IAT) techniques and emotional facial expressions analyses may contribute to the assessment of user experience (UX) during and after website navigation. These techniques have been widely and positively applied in assessing customer experience (CX); however, little is known about their simultaneous application in the field of UX. As a specific context, the experience raised by different websites from two well-known automotive brands was compared. About 160 Italian university students were enrolled in an online experimental study. Participants performed a Brand Association Reaction Time Test (BARTT) version of the IAT where the two brands were compared according to different semantic dimensions already used in the automotive field. After completing the BARTT test, the participants navigated the target website: 80 participants navigated the first brand website, while the other half navigated the second brand website (between-subject design). During the first 3 min of website navigation, emotional facial expressions were recorded. The participants were asked to freely navigate the website home page, look for a car model and its characteristics and price, use the customising tool, and in the end, look for assistance. After the website navigation, all the participants performed, a second time, the BARTT version of the IAT, where the two brands were compared again, this time to assess whether the website navigation may impact the Implicit Associations previously detected. A traditional evaluation of the two websites was carried on by means of the classic heuristic evaluation. Findings from this study show, first of all, the significant results provided by neuromarketing techniques in the field of UX, as IAT can provide a positive application for assessing UX played by brand websites, thanks to the comparison of eventual changes in time reaction between the test performed before and after website navigation exposure. Secondly, results from emotional facial expression analyses during the navigation of both brand websites showed significant differences between the two brands, allowing the researchers to predict the emotional impact raised by each website. Finally, the positive correlation with heuristic evaluation shows that neuromarketing can be successfully applied in UX.

## Introduction

Advances in technology, digital transformation, cost pressure, and the emergence of new channels have considerably changed the way customers shop and interact with brands (Gauri et al., 2016; Lemon and Verhoef, 2016; Bolton et al., 2018; Grewal et al., 2018; Lee, 2020). Furthermore, the challenges faced during the COVID-19 pandemic have brought companies to rethink their business models (Boudet et al., 2020). Today, customers are following a cross-channel customer journey rather than a linear path to purchase (Harris et al., 2020), and this big shift in consumer behaviour transforms them from buyers to users, moving the focus on the customer, and user experience (UX) (Sheth, 2021). Therefore, classic research techniques primarily based on qualitative methods such as self-report measures and interviews, largely predominating in UX research (Pettersson et al., 2018), require development for new UX evaluation methods. This development can improve practicability and scientific quality (Vermeeren et al., 2010), leading to multidisciplinary research methods based on more objective data (Verhulst et al., 2019). Among them, neuromarketing represents an evolving field of scientific investigations that have shown valuable understanding of consumer behaviour and its links with emotions in perception and decision-making processes. This area combines theories and practises from fields of behavioural sciences, including neuroscience, psychology, and sociology, to determine the reasoning and patterns of choices of consumers. As defined by Ale Smidts, as the first definition of the term, neuromarketing is, “*the study of the cerebral mechanism to understand the consumer's behaviour in order to improve the marketing strategies”* (Stasi et al., 2018).

In the field of neuromarketing, the most significant techniques are based on eye tracking, electroencephalography (EEG), fMRI, psychophysiology, analysis of facial expressions, and reaction times (Gacioppo and Petty, 1985; Stasi et al., 2018). Although neuromarketing techniques have been largely applied to customer experience (CX) (Gacioppo and Petty, 1985; Klinčeková, 2016), none of these techniques are commonly applied to UX, except for eye-tracking and a few pioneering studies relying on neuro and psychophysiological measurements (Bender and Sung, 2021). Emotions are considered a key point in UX, as mentioned by Marc Hassenzahl, one of the most quoted researchers in UX and its hedonic impact: “Reformers of Human Computer Interaction (HCI), often stress that the old HCI is, in essence, cognitive (i.e., focused on memory, task, etc.), and that the future lies in emotions” (Hassenzahl, 2004). Jakob Nielsen, one of the most well-known researchers in UX, in 1990, provided a set of nine “usability principles,” enabling the identification of all main problems when using HCI interfaces, software, and websites. However, in this set of usability principles, none of them was addressing attention to the emotional impact played by all these kinds of experiences. This lack was filled in a few years later, when Nielsen updated his list of nine “usability principles,” adding a 10th one, labelled as “aesthetic design” (Nielsen, 1994b), thus recognising the importance of the emotional impact played by the digital experience. Nevertheless, the way to investigate this additional principle is still mainly based on the opinion of expert evaluators as deeper described later in this article, mentioning the heuristic evaluation procedure. Almost two decades ago, Don Norman, another of the most quoted researchers in the field of UX, highlighted, and explained the importance of “emotional design” in products and services (Norman, 2004). Although famous scientists highlighted the importance of this factor, there is a lack of scientific procedures to characterise and measure this specific domain in UX. For this reason, as already stated, there is the need of developing new methods allowing to assess, according to empirical procedures, the effects of emotional impact played by UX. The present research brings light to this specific topic, showing how the application of two neuromarketing techniques allows researchers to assess and rank different websites in terms of emotional responses. One technique is based on an implicit association test (IAT) in relation to emotional items presented before and after website navigation to verify whether navigation can change short-term associations, as no previous research tried to apply this method to evaluate website experiences. The other technique is related to affective responses in terms of facial expression analyses during navigation, as little is shown by scientific literature in the field of UX and website design. The simultaneous application of both techniques, together with a traditional one relying on heuristic evaluation, allows researchers to explore whether the use of neuromarketing methods can improve the scientific measurement of the emotional design of websites, widening the application of neuromarketing techniques from CX to UX.

## Related Research Work

The IAT helps researchers identify biases through reaction times and emotions and has been developed into a marketing-oriented variation known as the Brand Association Reaction Time Test (BARTT) to expand on the understanding of consumer behaviour. This technique can clarify how consumers value the brand by distinguishing biases among participants through their intentional efforts to conceal attitudes towards concepts. When using IAT for evaluating brand associations, the data collected can indicate the subject biases and determine hierarchies of products by analysing reaction time as well as latencies in the way participants associate concepts (Bercea, 2012; Gregg and Klymowsky, 2013). Greenwald showed the reliability of this technique in his early IAT experiments, regarding pleasant/unpleasant associations, showing that the delays of participants, or lack thereof, indicate bias in addition to the choices selected (Greenwald et al., 1998). In this regard, the importance of this method can be argued for marketing research as a means of determining the biases of the customers through their conscious choices to avoid displaying biases. These biases are shown distinctly through the response latencies in the association of the concepts. Beginning with the seminal work of Keller (1993) and Aaker (2009), the brand association became an important topic, supported by the demand of marketers to have clear guidelines of brands in their business and managerial decisions. However, the literature shows a lack of research, enabling to highlight how brand associations may be modified by communication activities, in particular to websites communication strategies. Some research experiments already presented significant results about methods to measure brand image based on the constellation of associations (Till et al., 2011; Schnittka et al., 2012; Camarrone and Van Hulle, 2019). Some other research projects showed how brand associations can be efficiently applied to advertising assessments (Janakiraman et al., 2009; Anderson and Simester, 2013; Caldato et al., 2020). However, no previous research tried to investigate how brand associations vary depending on stimuli represented by a website. Brand associations are frequently characterised by a static mental map; however, what happens when users are exposed to a website? Does the mental map vary accordingly? Furthermore, to generate strong brands, firms have to implement a set of positive associations around them (Till et al., 2011; Flight and Coker, 2016). Through marketing actions, firms can identify, strengthen, or alter the associations linked to their brands (Keller, 1993), changing their competitive placement. The experience of a brand and consequently, its associations can be directly shaped by firms or can be even transferred by other brands or factors (Keller, 2003). Quite well-known examples are brand endorsement, co-branding, and brand extensions of celebrities (Martini et al., 2016). In this vision, the experience of a brand may be transferred to another one, if there are some bonds linking them. These bonds can also be retrieved in the brands of competitors as some associations are shared among different brands operating in the same market business. Based on the “transfer property,” what happens when competitors attempt to strengthen brand associations shared by the company? All these questions are still awaiting a proper and empirical answer. One of the aims of this research project is to fulfil the lack of scientific literature in the field of website communication and UX, exploring the dynamics of brand associations after being exposed to a website experience. In this way, it is possible to verify whether such exposure may raise short-term variations in the power of brand associations, not only on a specific brand but also on a competing one that may share some similar associations. Last but not least, as the impact of webpages on brand perception can be classified as an application of reaction time techniques in testing marketing stimuli, some positive evidence addressing the feasibility of such an application about the digital experience is supported by few pioneering studies (Matukin et al., 2016; Matukin and Ohme, 2017). Many methods from neuroscience research have been adopted to be used in marketing research (Clement et al., 2013; Missaglia et al., 2017; Songa et al., 2019), such as the IAT, but little is known about the possibilities these methods have in the area of UX with the exeption of few pioneering studies that show how to derive emotions from user mouse behaviour (Yi et al., 2020). User experience encompasses how people experience things around them, including products, websites, and services (Bojko, 2013). The Nielsen Norman Group defines UX as providing what the customer needs without hassle, crafting products that are a joy to own or use, and the “*seamless merging of the services of multiple disciplines, including engineering, marketing, graphical and industrial design, and interface design”* (Nielsen and Norman, 2021). Inherently, UX is multifaceted and touches on various parts of the use of a service, system, or product (Quaglini, 2020). To aid in the understanding of UX, researchers have relied greatly on a neuromarketing technique, such as eye tracking. It is especially relevant in evaluating the UX of websites and interfaces because it grants researchers the visual perspective of a user and allows to establish the findability of specific calls to action (Mele and Federici, 2012; Fu et al., 2017). Two primary reasons researchers use eye tracking are that it is non-invasive and can help determine how a consumer reacts during his or her interactions with a web product or service. Eye movements from the user can be fixations and saccades, and the movements of the eyes can indicate emotions such as confusion when the eyes return to a previous point (Bergstrom and Schall, 2014, p. 55–57). This tool is significant to researchers pursuing information about how consumers experience websites because the eyes of a person are drawn to and remain in places that result in further thinking, and the action of looking at a subject directly requires little conscious effort as it is a more reflexive process (Bojko, 2013). Points the eyes of users are drawn to on a web page contribute to fixation patterns that eye tracking technology can record, and these data can be converted into gaze plots and heat maps to determine points of significant focus on a web page (Djamasbi, 2014). Understanding the user behaviour on websites informs us about the decisions he or she makes, including ones to navigate away from the web page due to clutter or disorganisation. If a website suffers from these and other problems, it may result in an exhaustive review, which often frustrates the user since it is a product of a website that is not user-friendly (Nielsen and Pernice, 2010, p. 376). By using eye tracking, researchers can determine how a user views a site and navigates good or bad website design to improve the design for better UX. Therefore, eye tracking is a common and useful tool for researchers of UX. However, eye tracking does not help researchers understand a user's comprehension of a subject nor does it help indicate how a participant emotionally engages with the material in question (Bojko, 2013). Even though eye tracking is a useful tool for neuromarketing research and UX research, it, alone, does not provide the entirety of data needed to create an effective website. The IAT helps to understand the comprehension and opinions of a user towards a brand/subject/service or product, such as a website. Understanding brand association in consumers is integral to determining brand equity. Brand associations, as explained by Keller (1993), can be partitioned into three categories: associations of positive or negative favorability, uniqueness, and strength of associations (Gattol et al., 2011). In addition to these three elements, there is the relevance of the association and how this connexion may or may not present as a motivating factor, and the number of associations the consumer has (Gattol et al., 2011). A consumer may have significant associations for a brand, influencing the likelihood of a purchase from that brand as well as potential brand loyalty. A study analysing brand association in relation to a focus of prediction showed that participants had a significant association of brand names with cake flavour and quality (Van Osselaer and Janiszewski, 2001). Analysis of brand associations clarifies brand equity, and the aforementioned study establishes brand associations can lead to the positive favorability of a brand. However, previous studies of brand association have not utilised the technique of the IAT to evaluate the website experience of a user. The present project applied, for the first time, the BARTT to assess the effects of the experiences of two websites and to verify whether this technique can provide significant results enabling to measure and compare the effects of different website designs in relation to both emotional and cognitive items. The appliance of this neuromarketing technique to UX and website design widens the range of neuromarketing from customer to UX, verifying whether digital experiences can change short-term associations.

Additionally, the understanding of facial expressions helps researchers comprehend the emotional engagement and experience of a user towards the stimuli he/she has been exposed to during the navigation of a website. Automatic facial expressions analyses, efficiently used in neuromarketing to evaluate optimal advertising spots (Lewinski et al., 2014b; Lewinski, 2015; Hamelin et al., 2017; Cherubino et al., 2019) or the level of engagement during social media interactions (Schreiner et al., 2019), could lead to additional insights into UX research to improve the effects in terms of emotional design (Small and Verrochi, 2009; Norman, 2013; Hamelin et al., 2017; Danner and Duerrschmid, 2018). Facial expressions are part of non-verbal communication, which has been highlighted for a long time in the scientific literature as enabling to bring important information aside from verbal expressions (Stewart et al., 1987; Puccinelli et al., 2010). In this study, the affective reactions to websites will be measured in a quantitative way by means of autonomic responses, namely facial expressions. This allows the researchers to overcome some of the limitations of the most used tools in UX research, where the evaluation of emotional impact is mainly based on qualitative methods such as interviews. The feasibility of this approach is widened when an automated tool is utilised, engaging commercially available, advanced, and unobtrusive software that catches and analyses facial expressions of emotions. This solution has been already used in many different contexts related to experimental research in consumer behaviour. There are already several scientific studies showing how the use of automated facial analysis of expressions provides positive results in assessing CX (de Wijk et al., 2012; He et al., 2012; Terzis et al., 2013; Danner et al., 2014; El Haj et al., 2017; Noordewier and van Dijk, 2019; Riem and Karreman, 2019; Meng et al., 2020). Recently, new pioneering studies presented by the scientific literature have shown the possibility to take advantage of face orientation aside from facial expression to predict the hedonic impact of the face presentation of models, as the facial orientation to the right-side significantly predicts with a more negative evaluation, while on the opposite, face orientation towards left side significantly correlates with a positive evaluation of the models' face presentation (Park et al., 2021). Facial expressions reveal affective states defined, for instance, in EMFACS-7 (Friesen and Ekman, 1978) and thus possibly predict related behaviour and attitude modification (Kulczynski et al., 2016). Facial expressions of emotions are universal sequences of facial muscle contractions associated with the emotional state of the person. The neuro-cultural theory of emotion, developed by Paul Ekman (e.g., Ekman, 1972; Ekman and Cordaro, 2011), defines facial expressions of emotion as discrete, innate, and culturally independent. According to other studies, there is a two-way connexion between facial expressions and emotion regulation (Cole, 1986; Izard, 1990; Gross and Thompson, 2007; Gross, 2014). Therefore, in studying facial expressions, it is difficult to establish causal relationships between facial non-verbal behaviour and interpretations assigned to them—emotions. Emotions do cause facial expressions (“I feel happy, so I smile”), but facial expressions also cause emotions (“I smile and it makes me happy”). Any causal relationship between smiling and perception of the website has not been established in the UX context. Smiling or laughing may indicate liking for the website and, therefore, greater effectiveness of the website. Analysing facial expressions and user reactions to website interfaces identifies potential frustrations that can be improved for future users (Branco, 2006). Methods of facial expressions evaluation based on automatic software analyses further the understanding of the interaction of a user with one interface over another (Andersen et al., 2014), as well as the overall experience of the user with digital tools and resources (Liu and Lee, 2018). To understand UX based on emotions and facial expressions, the participants completed a series of tasks while sitting in front of a traditional PC equipped with a camera, allowing the software to measure the emotional reactions they had while interacting with a website, as this approach has been previously explored with positive results from pioneering studies (Hazlett, 2003). The technique of facial expression analysis has been used little by researchers of UX (Branco, 2006; Munim et al., 2017) despite its value in clarifying the frustration and joy of users during their interactions. According to Hancock et al., “Hedonomics,” (Hancock et al., 2005) defined as “the promotion of pleasurable human-machine interaction” by its creators, it is possible to highlight the key role of the so-called “emotional design” (Norman, 2004) as a fundamental factor in UX. The present research aims to explore whether the automatic facial expressions analyses may provide useful information related to the emotional reaction raised by website experiences. This research can expand the use of automatic facial expressions, helping professionals in measuring the effects of website emotional design according to more empirical procedures.

In conjunction with the two above techniques described, we integrated traditional heuristic evaluation (Nielsen and Molich, 1990; Nielsen, 1992) performed by five experts from the UX field. Combining traditional heuristic evaluation with innovative techniques based on reaction time and facial expression analyses can allow to explore whether the results from classic qualitative method based on heuristic evaluation converge or contrast with findings emerging from the use of quantitative methods based on facial expression analyses and reaction time measures. In the case of convergence, it may be possible to envisage a further integration of these innovative quantitative methods in UX research. On the opposite, in the case of divergence or contrast, it may be possible to understand whether these two different approaches are measuring different phenomena of UX. Jakob Nielsen developed the heuristic evaluation method together with usability consultant Rolf Molich in 1990 due to their many years of experience in teaching and consulting about usability and UX. As defined by the two authors, “there are four main methods to evaluate a user interface: formally, by some analysis techniques; automatically, by a computerised procedure; empirically, by experiments with test users; and heuristically, by simply looking at the interface and passing judgment according to one's own opinion” (Nielsen and Molich, 1990). In particular, the authors reported that “most user interface evaluations are heuristic evaluations, but almost nothing is known about this kind of evaluation since it has been seen as inferior by most researchers.” For this reason, they presented four experiments, enabling to derive a small set of nine “basic usability principles,” performed by at least three different professionals, enabling to identify all main problems. Few years later, Nielsen refined the heuristics based on a factor analysis of 249 usability problems (Nielsen, 1994a) that allowed the definition of a set of heuristics with maximum explanatory power, resulting in this revised set of heuristics that are used today by most professionals and organisations for user interface design (Nielsen, 1994b): visibility of system status; a match between system and the real world; user control and freedom; consistency and standards; error prevention; recognition rather than recall; flexibility and efficiency of use; aesthetic and minimalist design; help users recognise, diagnose, and recover from errors; help and documentation. Before this work, the guidelines were so many that a professional could need a lot of time before accomplishing it. For instance, Smith and Mosier's guidelines for designing user interface software have 944 items and remain one of the largest collections of publicly available user interface guidelines (Smith and Mosier, 1986). Another set of research-based heuristics has been proposed by Gerhardt-Powals (1996) to provide an alternative to Nielsen and Molich's list. Theoretically, all heuristics proposed to share the same purpose to established usability standards that, if enhanced, can provide a better UX about products or services. Unfortunately, “usability problems” are often identified by means of qualitative methods, relying on the opinion of expert evaluators (Catani and Biers, 1998). On one side, part of the problem could be explained considering that usability professionals have their own favourite sets of heuristics; on the other side, the problem is that there is not a research-based set of heuristics shared by the scientific community and based on international consensus. Moreover, the scientific literature addressed the need to update the heuristics provided many years ago: “with the rapid expansion and growth of technology in the last 20 years, Nielsen's 10 usability heuristics may need an update to remain consistent with modern usability problems” (Gonzalez-Holland et al., 2017). The present research study used a version of a heuristic evaluation set with 247 heuristics related to usability problems identified by Nielsen and revised specifically for website experience in the modern context, used in the professional field (Travis, 2017). The heuristic evaluation has been provided by five different professionals to establish whether a traditional and most-used method in UX may support findings from facial expressions analyses and reaction times techniques.

The inclusion of classic methods like heuristic evaluation with innovative techniques from the Neuromarketing field based on facial expression analysis and IAT helps to understand whether or not the combination of these different approaches may widen the insights on how UX is affected by the emotional design shaping websites contents and interactions.

Finally, both the BARTT/IAT and facial expression analysis have unique benefits in the current COVID-19 pandemic as they are reliable methods of obtaining information that can be collected and recorded without in-person interactions, taking complete advantage of a remote setting. The participants used a personal computer equipped with its camera to provide their facial recordings during the tasks assigned, releasing the needed data, enabling them to perform an automatic facial expression analysis. The IAT also only requires the use of a personal computer to be accomplished. Both parts of the experiment can be administered by the researcher through a video call or even an audio call, eliminating any need to meet all the participants in person. Due to the global pandemic, the need for health and safety of all those involved in the study was a high priority, so we relied on technology and internet connexion to acquire both accurate and safe data.

Regarding the subject of our experiment, we chose automotive sites from two American brands due to the impact of the pandemic on this industry. This research is intended to investigate how the brands might take advantage of innovative insights for developing new digital strategies to overcome the crises raised by the COVID-19 pandemic and improve automotive sales through their websites.

## Materials and Methods

The study was conducted between October 2020 and December 2020, and a sample consisting of 160 students (80 men, 80 women; mean age, 23 ± 4) was recruited from the Catholic University of Milan. One criterion was established to qualify the sample: The participants had to be in-market for a car and intended to purchase it within an appropriate time frame of 2–3 months. In the event that the website proposed cars beyond their budget, we asked them to identify themselves with a potential buyer. The participants who had already made their minds about exactly which car they were going to buy were removed from the sample to exclude the possibility that the participants might have already exhausted their capacity for exploration and evaluation of the website. The fact that all the participants are university students provides the limit that all results are representative of this specific population, and further research with broader samples in terms of age range and low/high skills in information technology may establish whether the results here presented can be representative of the whole general population. All the participants were required to have an internet connexion and a personal computer equipped with a webcam. The minimum definition resolution required to participate in the test is a standard high-definition of 1,280 × 720 pixels (HD Ready or 720 pixels). Two websites from the automotive field have been selected to perform a comparative test: Ford and Tesla (version exposed in 2020). This study was performed remotely by utilising software, including iCode, for online IAT provided by NEUROHM and FaceReader 8.1 software from Noldus for emotional facial expression analysis. All of the participants completed an online, pre-test survey that related to the application of IAT.

iCode, an online platform in the field of reaction time recording, was used to assess the speed in providing their answers from all the participants in this project. iCode accurately reflects the attitudes of the participants by using a two-part calibration process to analyse response time (iCode., 2019). Part one of the calibration process of iCode uses motoric tasks to establish the movement speed and familiarity with the device of each participant (iCode., 2019). The calibration of iCode consists of pressing the answer buttons without any cognitive load. It also serves as a tutorial for respondents as it makes them familiar with using the scale. Part two of the calibration process of iCode tests how fast the participants read statements of different lengths. Each participant was given a statement and one answer button to press when he/she finished reading the statement. The influence of statement length on corresponding response times is minimised, allowing statements of different lengths to be compared (iCode., 2019). iCode uses Neurohm's Confidence Index to ensure accuracy and helps researchers determine the emotional certainty of participant opinions. However, to perform a statistical analysis according to indications shared in the scientific literature, results from this project rely on raw data expressed in milliseconds recorded by the iCode online platform.

Facial expressions of the participants were recorded during web page navigation and processed in post-test using FaceReader, version 8.1, from Noldus (Noldus, 2014; Loijens and Krips, 2019). Objective facial measurements were used to capture reactions to website exposure (Den Uyl and Van Kuilenburg, 2005). This system uses a three-layer neural network that automatically identifies and examines facial expressions of emotions in human beings (Den Uyl and Van Kuilenburg, 2005). It detects and classifies facial expressions both from pictures and videos into one of the following basic emotions: happy, sad, angry, surprised, scared, disgusted, contempt, and neutral (Ekman, 1972). Facial expressions, like happiness, sadness, etc., are examined in FaceReader on a frame-by-frame method. This is since basic emotions can usually be expressed in full within a single frame (snapshot) of the face. However, there exist many more complex affects, which are not completely expressed with a single instance but rather, over a longer amount of time. These longer temporal facial affects are called “*affective attitudes*.” With the release of FaceReader 7.1, the analysis of three commonly occurring affective attitudes, namely: interest, boredom, and confusion has been introduced. Unlike regular facial expressions, these affective attitudes are computed over a time window (typically from 2 to 5 s), rather than a frame-by-frame method. Therefore, the intensity of the affective attitude at any point in time of analysis does not just depend upon the current analysis of the face but also on the last 2 pr 5-s history of facial analysis. In addition, some of these affective attitudes also take into account certain additional facial cues like nodding or head shaking, which are also internally computed over the analysis history. The literature on the affective attitudes is still exploring the accuracy of these additional metrics (Borges et al., 2019; Hirt et al., 2019); we provide here results related to confusion, as particularly useful to evaluate the impact of website experience here considered, as previous research showed positive results in considering subtle expressions (Salgado-Montejo et al., 2015).

First, FaceReader detects a face using the so-called “Active Template Method.” Second, the software builds a virtual, super-imposed 3D “Active Appearance Model” of the face, featuring nearly 500 distinctive landmarks. The third step measures the intensity and probability of facial expressions, enabling basic emotions to be computed (Van Kuilenburg et al., 2005). The neural network of the system has been trained, taking advantage of a high-quality correlation of approximately 10,000 images that were manually annotated by real human expert coders. The average scores of performances reported are 89% (Den Uyl and Van Kuilenburg, 2005; Van Kuilenburg et al., 2005) and 87% (Terzis et al., 2013). We consider in this study in the present project results only about “happiness,” as the accuracy of this specific emotion is the highest in comparison to all other emotions according to the scientific literature (Lewinski et al., 2014a,b; Stöckli et al., 2018; Dupré et al., 2020). Although FaceReader can analyse offline videos, our study required the participants to have a live webcam to classify facial expressions in real time. FaceReader contains five different face models that are used to find the best fit for the face that is going to be analysed. The models include: (1) “General,” the default face model; (2) “Children,” the face model for children between ages 3 and 10 years old; (3) “East Asian,” the face model for people of East Asian descent (Zhi et al., 2017) e.g., Korean, Japanese, and/or Chinese; (4) “Elderly,” a model for participants 60 years of age and older; (5) “Baby FaceReader,” different software for infants between ages 6 and 24 months old. We set FaceReader to “General” for this study to account for the mean age (23) and nationality (Italian) of the participants.

We did not use FaceReader's Participation or Group calibration. Instead, we used “in the wild,” or spontaneous, facial expression data to predict real-world consumer responses. Facial expressions, often caused by a mixture of emotions, can occur simultaneously at high intensities (Loijens and Krips, 2019). Spontaneous facial expressions are, therefore, processed immediately after being recorded. This process works well for larger samples; thus, spontaneous facial expressions were ideal for our study of 160 participants. Spontaneous expressions can also provide a benchmark for comparisons between different algorithms (Küster et al., 2020). We relied on a minimum of 90% of accurate facial analysis through all FaceReader analyses detected for each participant: each participant has been exposed to the website for a total of 3 min, and his/her facial expressions have been recorded for a total of 180 s. Only recordings that FaceReader processed properly for at least 162 s were considered. However, results from the 16 participants (seven from the group assigned to the Tesla website and nine from the group assigned to the Ford website) were discarded due to the participants leaning into the camera, covering their faces, or otherwise interfering with the tracking and expression analysis of the FaceReader. We enrolled 16 additional participants, according to gender characteristics and willingness to purchase a car within the next trimester. In this way, the researchers have been able to make up for those discarded and checked in real time the quality of facial expression analysis to collect datasets of 80 participants for each group.

All the participants were guided by a researcher to complete the protocol steps of the study. Prior to completing the study, consent forms were sent to the participants *via* email. The implicit association pre-test was sent to the participants through an email link. The following statements were used in the pre-test: reliable (affidabile), passion (passione), I would like to own it (mi piacerebbe averla), comfortable (confortevole), innovative (innovativa), I would like to have it (mi piacerebbe averla), electric (elettrica), safe (sicura), traditional (tradizionale), affordable (accessibile). All statements were presented in association with the logo of the two brands: eighty participants undertook a “response latency” task, in which they were asked to respond “yes” or “no” to each brand/association pair. According to the model of Till et al. (2011), we consider the speed of response as an implicit measure of the association strength: the faster the response to the association, the stronger the association. We also recorded the number of explicit responses in terms of “yes” or “no,” as well as the speed of their responses, which are defined as “response latency.” Our procedure was based on the “Brand Association Reaction Time Task” (BARTT) script provided by iCode, which enables measurement of the frequencies and reaction times of opinions of the participants as to whether or not words are associated with the target brands, as described in Till et al. (2011). Based on the theoretical perspective described in the method section, our procedure was designed to find out the associations that are part of the immediate network of a brand and to provide an analysis of those associations in terms of their frequency and strength; regarding the “frequency,” it is defined as “the number of mentions over the associations to the brand”: as shown by Teichert and Schöntag (2010), the more respondents have similar associations, the higher the average node strength. Relating to the “strength,” it is defined as follows: “the latency of response to the brand associations” (Sanbonmatsu and Fazio, 1990). The faster the subjects responded to the target investigation, the stronger the association. For each brand (TESLA and FORD), we first calculated the “Frequency of Associations” (FoA) and, secondly, the “Strength of Associations” (SoA). Only the “yes” answers were considered both for FoA and SoA (Till et al., 2011).

The 160 participants were randomly assigned to either Ford or Tesla. About 80 participants navigated Ford's website, and the other 80 participants navigated Tesla's website.

The web page navigation process required the participants to complete four navigation steps. If a participant was unable to complete a task within the allotted time, the task was marked as failed. However, the participants were not penalised for failure and could proceed to the next step. The participants would signal to the researcher that they are ready to begin the task by showing a thumbs-up or waving. The participants would then use a webcam to record themselves completing the task. After the participants completed the four navigation tasks, all video recordings would be sent to the researcher for analysis.

**Figure 1:**
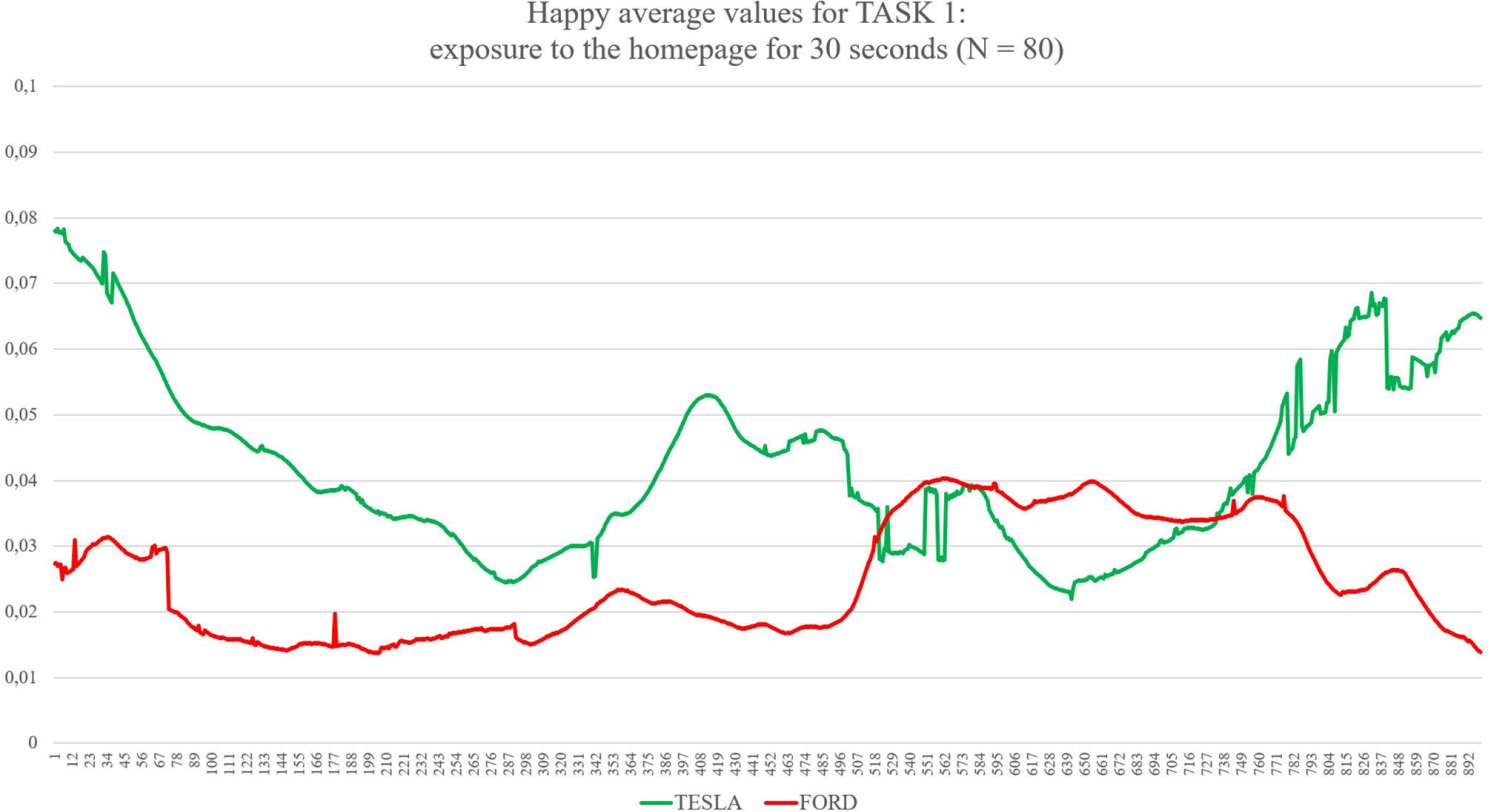
The graph shows continuous values of Happiness for both Ford and Tesla during Task 1 (exploration of the homepage for 30 s). The average levels of happiness are higher during the exploration of the Tesla website compared with Ford. On the X axis, time is expressed in 30 samples per second for a total of 900 samples, corresponding to 30 s as the total amount of time exposure. On the Y axis, values of happiness are expressed between 0 (no happiness expressed by the face) and 1 (highest intensity of happiness expressed by the face).

**Figure 2:**
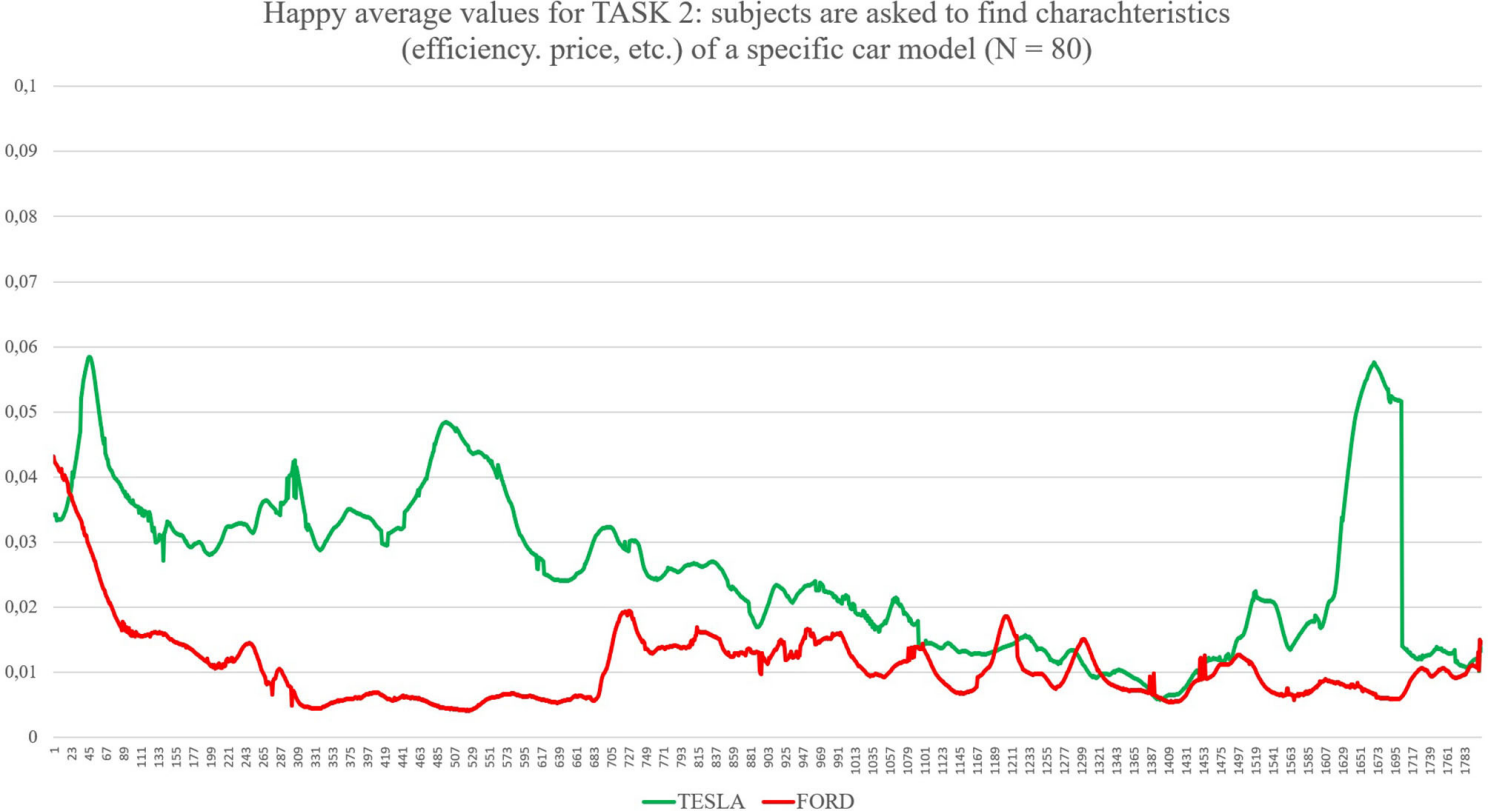
The graph shows values for Happiness for both Tesla and Ford during Task 2 (subjects were asked to look for characteristics of a specific car model). On the X axis, time is expressed in 30 samples per second for a total of 1,800 samples, corresponding to 60 s as the total maximum amount of time exposure to the website during this task. On the Y axes, values of happiness are expressed between 0 (no happiness expressed by the face) and 1 (highest intensity of happiness expressed by the face).

**Figure 3:**
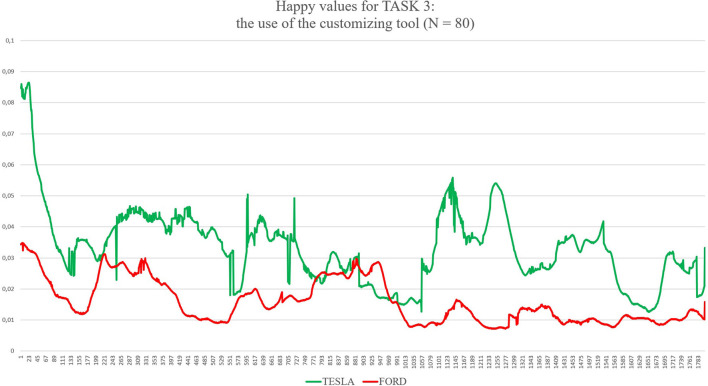
The graph shows values for Happiness both for Tesla and Ford during Task 3 (subjects were asked to use the customising tool). On the X axis, time is expressed in 30 samples per second for a total of 1,800 samples, corresponding to 60 s as the total maximum amount of time exposure to the websites for this task. On the Y axes values of happiness are expressed between 0 (no happiness expressed by the face) and 1 (highest intensity of happiness expressed by the face).

**Figure 4:**
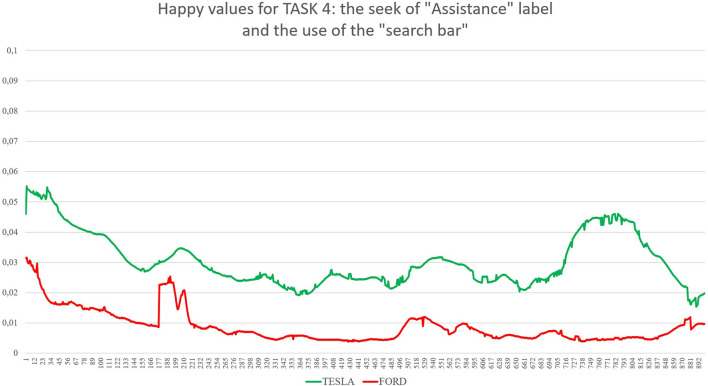
The graph shows values for Happiness for both Tesla and Ford during Task 3 (subjects were asked to use the customising tool). On the X axis, time is expressed in 30 samples per second for a total of 900 samples, corresponding to 30 s as total maximum amount of time exposure to the websites for this task. On the Y axis, values of happiness are expressed between 0 (no happiness expressed by the face) and 1 (highest intensity of happiness expressed by the face).

Task 1, the first impression test, exposed each group to its randomly assigned website homepage for 30 s. The participants were asked to only scroll and avoid clicking when interacting with the homepage of the website (as shown in Figure 1). Task 2 gave participants 1 min to look for a specific model and its functional characteristics, such as acceleration, maximum speed, efficiency, and price (as shown in Figure 2). The participants randomly assigned to Tesla were asked to find the “Model X,” and the participants randomly assigned to Ford were asked to find the “New Explorer.^1^” The “Model X” and “New Explorer” are comparable in price. Task 3 gave the participants 1 min to customise a specified vehicle model. The participants randomly assigned to Tesla designed a “Model 3,” while the participants randomly assigned to Ford customised a “Mustang Bullitt^2^” (as shown in Figure 3). Task 4 asked both groups to envisage the need for assistance to find information on electric battery packs (as shown in Figure 4). For the task to be successful, the participants were required to utilise the search bar. Thus, the participants who found the information without the search bar failed the task. All the participants from both groups were given 30 s to complete Task 4.

After completing the four tasks, both groups were instructed to complete the implicit association post-test. All 160 participants completed the same implicit association post-test, regardless of their assigned company. During the post-test, the participants were asked to apply their perception of the two homepages after UX. The participants were asked if they associate the homepage of Tesla and/or Ford with the following perception characteristics: Trust and Credibility, Easy Navigation, Pleasant Visual Design, Promotion, Clear Information, and Assistance. All these items were chosen from the dimensions used in heuristic evaluation in order to compare results from both techniques, with the exception of “Promotion,” which was selected as there is a strong difference between the FORD website, rich of promotions, and TESLA website, where there are no promotions. Screenshots of the homepages were used to provide the participants with a visual aid, and once again, they had to choose between a “yes” or “no” response in associating each item.

The participants were then asked to complete a five-item questionnaire with a Likert scale of 9 points. The following questions were asked: How did you evaluate the website that you navigated (from ≪0≫ = negative evaluation; to ≪9≫ = positive evaluation)? To what extent did you like the homepage of the website (from ≪0≫ = I did not like it at all; to ≪9≫ = I liked it a lot)? In your opinion, was it easy to find characteristics (such as max speed, acceleration, efficiency, etc.) and price (from ≪0≫ = very hard; to ≪9≫ = very easy)? Was it easy to use the car customisation tool (from ≪0≫ = very hard; to ≪9≫ = very easy)? Was it easy to find the search bar for customer service/assistance (from ≪0≫ = very hard; to ≪9≫ = very easy)?

**Figure 5:**
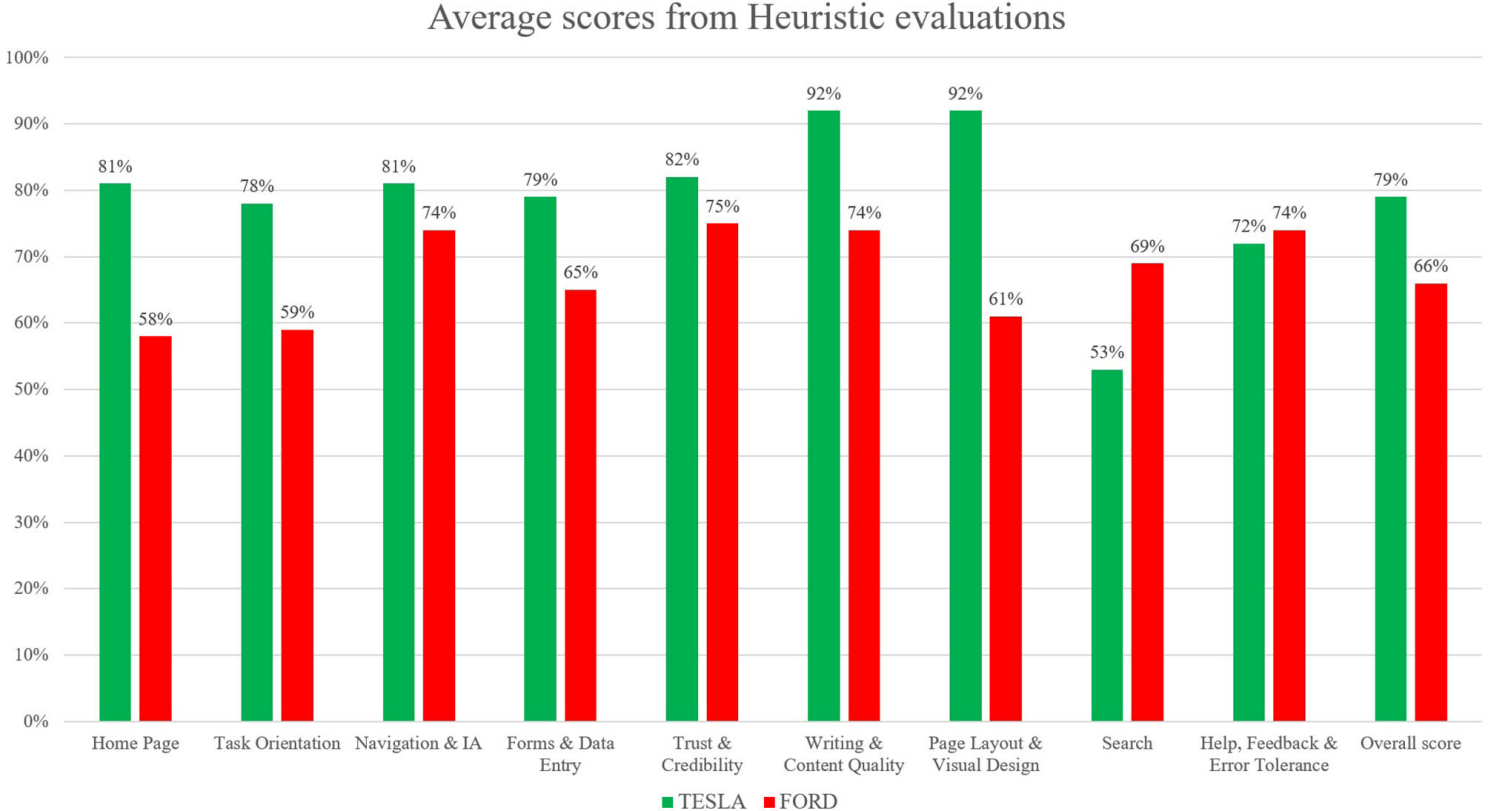
Results from the heuristic evaluation analysis performed by five judges as professional experts in the field of User Experience (UX). The heuristic evaluation was performed in order to provide a qualitative evaluation about a traditional technique applied to assess UX; no statistical analysis was applied to the collected data. The overall scores show that the Tesla website provides a better UX in comparison with the Ford website (Tesla scored 79%, while Ford 66%). In particular, the dimensions of: “Home Page,” “Writing and Content Quality,” “Page Layout and Visual Design,” and “Task orientation” show the highest gap between the two websites.

Separate from the questionnaire, five expert professionals in the field of ergonomics and UX performed the heuristic evaluation from both websites (see Figure 5). This evaluation helps to identify usability scores in the following dimensions: Home Page (20 heuristics to evaluate the usability of the homepage, partially covering the sixth Nielsen principle “recognition rather than recall” and partially covering the fourth Nielsen principle “consistency and standard”), Task Orientation (44 items aimed to assess the ability of the website in supporting the tasks of users, covering the fifth Nielsen principle “Error prevention”), Navigation and Information Architecture (29 questions aimed to evaluate user navigation, correlating with the third principle from Nielsen's heuristics, “user control and freedom”), Forms and Data Entry (23 items, partially covering the fourth Nielsen principle “consistency and standard” and partially covering the fifth Nielsen principle “error prevention”); Trust and Credibility (13 items, partially covering the first Nielsen principle “visibility of status”), Writing and Content Quality (23 items, partially covering the second Nielsen principle “a match between system and real world”), Page Layout and Visual Design (38 items, covering the eight Nielsen principle “aesthetic and minimalist design”), Search (20 items, partially covering the seventh Nielsen principle “flexibility and efficiency of use”), Help, Feedback, and Error Tolerance (37 items, covering the ninth Nielsen principle “helping users recognise, diagnose, and recover from errors”). The heuristic evaluation portrays the qualitative assessment of UX by means of a well-established procedure (Nielsen and Molich, 1990) where each score is derived by a standardised procedure based on the answers to 247 questions, covering all the dimensions mentioned above, where professionals can choose one of the following “answers”: “+1” (that means the website respects the guidelines), “−1” (the website does not respect the guidelines), and “0” (The website respects the guidelines in part only). These five expert professionals did not participate in the IAT pre- and post-test, and their facial expressions were not recorded.

## Results

The main output of FaceReader classifies facial expressions from the participants according to intensity. Facial expressions are valued between 0 and 1, where 0 denotes an absent expression, and 1 indicates a fully present expression. FaceReader also calculates valence, which indicates whether the emotional state of each participant is positive (happy) or negative (sad, angry, or disgusted). Valence is equivalent to the intensity of positive expression minus the highest intensity of the three negative expressions. FaceReader calculated arousal, indicating whether the participant is active (+1) or not active (0). Arousal is based on the activation of 20 Action Units (AUs) of the Facial Coding System (FACS).

First, the *t*-test (two-tailed) on results related to the automatic detection of the facial expression of happiness as an emotional reaction during the navigation of the two websites showed significant differences between the two groups (see Table 1): for Task 1 (statistic = −2.50, *p* = 0.015), where Tesla elicited significant higher emotional expressions of happiness in comparison to Ford website during the exploration of the home page for the first 30 s; for Task 2 (statistic = −2.51, *p* = 0.014), where Tesla website showed higher induction of happier facial expressions in comparison to a website from Ford while users explored the characteristics of cars models, such as speed, acceleration, price, and so forth; for Task 3 (statistic = −2.04, *p* = 0.046), where Tesla website elicited higher emotional facial expressions of happiness in comparison to Ford website while user used the car-customising tool; for Task 4 (statistic = −3.23, *p* = 0.002), where Tesla website induced increased facial expressions of happiness in comparison to Ford website, while users searched for the information related to electric recharge of cars equipped with an electric battery pack. Additionally, the applied results show confusion as an emotional reaction during the exploration of both websites (Tesla vs. Ford). Concerning the automatic detection of facial expressions, the *t*-test showed significant differences between Tesla and Ford only for Task 4 (statistic = −2.81, *p* = 0.008).

**Table 1:** The average values for Happiness and Confusion for both groups (Ford and Tesla) during the four navigation tasks.

	**Task 1**		**Task 2**		**Task 3**		**Task 4**	
**Emotion**	***Ford***	***Tesla***	***Ford***	***Tesla***	***Ford***	***Tesla***	***Ford***	***Tesla***
Happiness	0.0112^*^	0.0451^*^	0.0117^*^	0.0232^*^	0.0114^*^	0.0381^*^	0.0104^*^	0.0310^*^
Confusion	0.0098	0.0043	0.0088	0.0106	0.0139	0.0111	0.0039^*^	0.1001^*^

*Values bolded with an asterisk indicate significant differences*.

Statistical analyses on reaction times were performed, as the first step, regarding the FoA. Descriptive statistics for both brands (Tesla vs. Ford) and relative associations (i.e., reliable, passion, comfortable, innovative, I would like to have it, electric, safe, traditional, and accessible) were calculated and reported in Table 2 (for 80 subjects exposed to the Tesla website) and Table 3 (for 80 subjects exposed to the Ford website). The dataset consisted of “Yes” answers from 160 participants,10 brand associations (i.e., reliable, passion, it makes me free, comfortable, innovative, I would like to have it, electric, safe, traditional, and accessible), and two brands (i.e., Tesla and Ford) recorded before website experience and after website experience. No significant results emerged from FoA dataset analyses.

**Table 2:** Frequency of Associations (FoA) expressed in percentage of the number of “yes” answers over the total sample (80 subjects exposed to the Tesla website).

	**Ford**	**Tesla**	**Ford**	**Tesla**
	**(Pre-test)** **(%)**	**(Pre-test)** **(%)**	**(Post-test)** ** (%)**	**(Post-test)** ** (%)**
Reliable	96	97	98	88
Passion	48	48	51	76
It makes me free	35	56	39	69
Comfortable	92	91	90	89
Innovative	35	96	38	99
I would like to have it	41	58	48	84
Electric	35	95	37	99
Safe	96	97	95	91
Traditional	91	13	95	9
Accessible	97	25	97	26

**Table 3:** FoA expressed in percentage of the number of “yes” answers over the total sample (80 subjects exposed to the Ford website).

	**Ford**	**Tesla**	**Ford**	**Tesla**
	**(Pre-test)** **(%)**	**(Pre-test)** **(%)**	**(Post-test)** **(%)**	**(Post-test)** **(%)**
Reliable	94	94	97	90
Passion	51	79	48	80
It makes me free	36	57	44	59
Comfortable	90	90	96	92
Innovative	34	93	55	94
I would like to have it	46	61	47	60
Electric	36	94	73	95
Safe	94	95	88	96
Traditional	95	11	87	10
Accessible	96	23	88	25

*No differences between the group made by Tesla and Ford users were observed*.

As a second step, statistical analyses were performed in the SoA: in this case, only the “Yes” answers were considered (when subjects choose “Yes” to express a positive association between a brand, either Tesla or Ford, and dimensions, namely: reliable, passion, it makes me free, comfortable, innovative, I would like to have it, electric, safe, traditional, and accessible). Before proceeding with the analysis, we removed outliers that were defined as response latencies below 300 ms and above 3,000 ms (Greenwald et al., 1998). No differences between the group made by Tesla or Ford users were observed. Outliers were identified and removed according to the threshold, which is typically employed with analysis involving reaction times. Table 4 shows the SoA for the 80 subjects exposed to the Ford website, while in Table 5, the 80 subjects were exposed to the Tesla website. As a third step, *t*-test statistical analyses performed on SoA data from the *Ford* dataset for each association were examined; analyses revealed significant differences between pre- and post-test for the following associations: reliable, it makes me free, innovative, electric, traditional, and accessible (as shown in Table 4). As a fourth step, a *t*-test performed on SoA data from the *Tesla* dataset for each association was considered; results revealed significant differences between pre- and post-test for the following associations: reliable, passion, it makes me free, comfortable, I would like to have it (as shown in Table 5). As a fifth step of the analysis, a comparison has been considered between results from Ford and results from Tesla: the comparison shows that there are two associations shared by both brands: “reliable” and “it makes me free.” However, the two brands differ regarding all other associations. The experience on the Ford website has been able to increase the associations of: “innovative,” “electric” (these two are related to technological issues), “traditional” (related to the perception of a brand considered as a long-established presence in the automotive market), and “accessible” (perception of the Ford website as an experience enabling to convey information, allowing to evaluate the brand as more affordable); while the experience on the Tesla website has been able to increase the associations of: “passion,” “comfortable,” “I would like to have it” (all these three dimensions deal with emotional reactions: “passion,” as a powerful feeling barely controllable by rational thinking; “comfortable,” the Tesla website has been able to convey information related to a car that is more prone to providing physical ease and pleasant relaxation while using it; “I would like to have it” deals with the desire of owning that car, once again highlighting the feeling, worthy, or unworthy, that impels to the attainment or possession of something that is, in reality or in imagination, able to bring satisfaction and/or enjoyment).

**Table 4:** Strength of Associations (SoA) expressed in milliseconds of the number of “yes” answers over the total sample (80 subjects exposed to the Ford website).

	**Ford**	**Tesla**	**Ford**	**Tesla**
	**(Pre-test)**	**(Pre-test)**	**(Post-test)**	**(Post-test)**
Reliable	2,269	2,323	**1,982** (***p*** **= 0.046)**	**2,099** (***p*** **= 0.014)**
Passion	2,361	2,314	2,166	2,182
It makes me free	2,327	2,459	**1,952** (***p*** **= 0.039)**	2,307
Comfortable	2,379	2,351	2,120	2,241
Innovative	2,358	2,139	**2,109** (***p*** **= 0.031)**	2,113
I would like to have it	2,419	2,297	2,198	2,167
Electric	2,534	2,187	**2,156** (***p*** **= 0.021)**	2,016
Safe	2,269	2,318	2,113	**2,155** (***p*** **= 0.019)**
Traditional	2,369	2,041	**2,165** (***p*** **= 0.012)**	1,943
Accessible	2,304	2,116	**2,211** (***p*** **= 0.047)**	1,956

*Values in bold designate a significant difference between pre- and post-tests*.

**Table 5:** SoA expressed in milliseconds of the number of “yes” answers over the total sample (80 subjects exposed to the Tesla website).

	**Ford**	**Tesla**	**Ford**	**Tesla**
	**(Pre-test)**	**(Pre-test)**	**(Post-test)**	**(Post-test)**
Reliable	2,201	2,361	**2,039** (***p*** **= 0.043)**	**2,157** (***p*** **= 0.049)**
Passion	2,269	2,591	2,061	**2,105** (***p*** **= 0.029)**
It makes me free	2,477	2,548	2,186	**2,218** (***p*** **= 0.026)**
Comfortable	2,282	2,351	2,187	**2,143** (***p*** **= 0.032)**
Innovative	2,279	2,322	2,245	2,130
I would like to have it	2,239	2,521	2,113	**2,124** (***p*** **< 0.001)**
Electric	2,083	2,105	2,264	2,007
Safe	2,247	2,349	2,097	2,098
Traditional	2,233	2,367	2,209	2,101
Accessible	2,196	2,313	2,309	2,109

*Values in bold revealed a significant difference between pre- and post-tests*.

Statistical analyses were performed on the collected data on the short survey exposed after the website navigation, in relation to the perception of both Tesla and Ford websites in terms of reaction time (the six items exposed were: “Trust and Credibility”; “Easy Navigation”; “Pleasant Visual Design”; “Promotion”; “Clear Information”; “Assistance”). The *t*-test showed a significant difference between the two groups (Tesla vs. Ford) for one item only: “Pleasant Visual Design,” where the reaction time is faster for subjects who navigated the Tesla website in comparison to Ford (see Table 6). Finally, statistical analyses were performed in the last data collected concerning the short survey, exploring the judgments expressed by each participant who navigated the website about the navigation [the five items investigated were: “Do you like the website?”; “Do you like the Homepage?”; “Was it easy to find car characteristics and price?”; “Was it easy to use the customisation tool?”; “Was it easy to find assistance (use of the search bar)?”]; The *t*-test showed a significant difference between the two groups (Tesla vs. Ford) for all items (see Table 7). Results from heuristic evaluations performed by five different expert professionals show that, except for the dimension of “Help, Feedback, and Error Tolerance,” where the two websites scored very similar values (74% for Ford and 72% for Tesla), and except for the dimension of “Search,” where the Ford website scored on average a greater value in comparison to Tesla (69% for Ford and 53% for Tesla), all the other dimensions are showing, on average, a higher score for Tesla in comparison to the Ford website (see Table 8); in particular, the highest difference is for the dimension of “page layout and visual design” (where Tesla scored, on average, 92% while Ford 61%); “Home Page” (where Tesla scored, on average, 81% while Ford 58%); “writing and content quality”(where Tesla scored, on average, 92% while Ford 64%); “task orientation” (where Tesla scored, on average, 78% while Ford 59%). The overall scores from heuristic evaluations indicate that the Tesla website seems to provide an overall better UX in comparison to the Ford website (Tesla scored 79% while Ford 66%).

**Table 6:** The final survey values (expressed in milliseconds) about reaction time expressed in milliseconds.

	**Ford HP (Post-test)**	**Tesla HP (Post-test)**
Trust and credibility	2,362	2,430
Easy navigation	2,314	2,352
Pleasant visual design	2,441	**2,171** (***p*** **= 0.047)**
Promotion	2,250	2,352
Clear information	2,263	2,198
Assistance	2,054	2,248

*The reaction time is compared and analysed for each item. Values in bold indicate a significant difference*.

**Table 7:** Results from the final survey expressed by means of average scores for each item (from 1 to 9).

	**Ford HP (Post-test)**	**Tesla HP (Post-test)**
Do you like the website?	6.5	**7.3** (***p*** **< 0.001)**
Do you like the Homepage?	6.4	7.9 (***p*** **< 0.001)**
Was it easy to find car characteristics and price?	6.0	**6.9 (*****p*** **= 0.022)**
Was it easy to use the customisation tool?	6.1	7.2 (***p*** **<** ***p*** **< 0.001)**
Was it easy to find assistance (use of the Search bar)?	7.4	3.7 (***p*** **< 0.001)**

*Values in bold revealed a significant difference between the two groups (80 subjects navigated Ford website and 80 subjects Tesla website). At the end of website navigation, all subjects were asked to fill in a brief online questionnaire, providing their responses by means of a 9 point Likert scale (from 1 = “not at all”; to 9 = “A lot”). For instance, the first question is: “Do you like the website?”. Subjects who navigated the Ford website scored on average 6.5 on a 9 points Likert scale, while the 80 subjects who navigated the Tesla website scored on average 7.3 on the same 9 points Likert scale: there is almost one point of difference, revealing a significantly higher level of satisfaction for Tesla website in comparison to Ford website*.

**Table 8:** Results from heuristic evaluations expressed by means of average percentage scores for each dimension (in bold, the highest differences between the two website average scores).

	**Ford (%)**	**Tesla (%)**	**Difference (%)**
Home page	**58**	**81**	**23**
Task orientation	**59**	**78**	**19**
Navigation and information architecture (IA)	74	81	07
Forms and data entry	65	79	14
Trust and credibility	75	82	07
Writing and content quality	**74**	**92**	**18**
Page layout and visual design	**61**	**92**	**31**
Search	**69**	**53**	**16**
Help, feedback and error tolerance	74	72	02
Overall score	66	79	13

## Discussion

The aim of this study was to examine whether the use of automatic facial emotional expression analyses and reaction time methods may broaden the assessment of UX in young adults by using novel integration of techniques that combine a variety of approaches based on self-report and heuristic evaluation coupled with software both for emotional facial detection and reaction time measurements recorded by means of an online quantitative procedure only. Data analysed indicated that the two groups, each exposed to one of the two websites of well-known American brands in the automotive industry, reacted in a significantly different way for all the methods considered. The Tesla website has been able to induce a stronger emotional reaction, according to all results. In terms of facial expressions, it elicited much higher expressions of happiness in all the tasks performed. Taking into account the results from heuristic evaluation where average scores for “web layout and visual design” and “homepage” are higher for the Tesla website in comparison to the Ford website, and taking into account results from the self-reports from all the participants enrolled in the research projects, showing significant differences in favour of the Tesla website in comparison to the Ford website, together with time reaction analyses for the item “Pleasant Visual Design” from the survey that displays significant faster response for the Tesla website, it is possible to claim a greater emotional impact played by the Tesla website in comparison with Ford. This pattern of better emotional performance is also supported by semantic dimensions investigated through reaction time technique too: they show that respondents perceived the Tesla website as conveying information, enabling to change implicit attitudes for “reliable,” “passion,” “freedom,” “comfortable,” and “desire to own it.” Taken altogether, all these dimensions are more related to emotion rather than functions or information about car performances and prices. At the same time, results show how participants are convinced Tesla is not a traditional brand and they do not believe it is an accessible car (as there are no significant differences for those two dimensions).

On the opposite, results from FORD show a less important emotional impact, not only in terms of facial expressions related to happiness, always at a lower level in all tasks accomplished on the FORD website but also for all other techniques considered. Heuristic evaluation from five expert professionals in the field or UX showed, on average, a decreased score (with the exception of the items of “Search” and “Help, Feedback, and Error Tolerance”) for the FORD website in comparison with the TESLA website. The final survey showed significantly decreased Likert scale scores for all items in comparison with FORD, except for the “Search bar” (we will consider that specific issue later here in this section). Finally, the dimensions investigated by means of reaction time analyses reveal that FORD websites have been able to convey information enabling to change implicit attitudes for “reliable,” “freedom,” “innovative,” “electric,” “traditional,” and “accessible.” Except for the dimension of “freedom,” all other items are more related to information cognitively conveyed by the website: innovation, and electric are the best examples, as the FORD website shows the latest innovation regarding the technology implemented in some models and the “electrification process” started by the company developed few hybrid models; in addition, in Task 3, the participants had to look for a model, the “Mustang Bullitt,” which also presented a version of a car model that is completely electric (the only one from FORD panorama of car models). The dimensions of “Traditional” and “Accessible” are more related to the general brand perception of an automotive organisation that appeared in the market a long time ago and to provide much more affordable models in comparison to Tesla, even if the models selected within tasks accomplished by experimental subjects were chosen according to a similar placement (a similar price range).

Considering the specific case represented by Task 4, it is possible to evaluate the emotional impact played by two different design choices more related to “information architecture.” TESLA shows the button “Assistance” as the 11th label of a vertical menu completely hidden in a hamburger menu located on the top-right side of the homepage: a user has to identify it (he/she has to know or understand that the three small horizontal lines on the top-right of the homepage are a sort of a small icon that represents a so-called “hamburger menu,” enabling to explode a menu only once requested) and click to open it on the right side of the screen. FORD shows the same call to action directly in the upper side of the homepage, as the fourth label of a horizontal menu composed of four labels in total, where the label “Assistance” is available at a first look. Data collected show which one of the two design solutions is preferable for users; this time, FORD seems to perform much better in comparison with TESLA. The heuristic evaluation average scores show better results for this specific function, and the survey brings a significant positive preference for FORD in comparison to TESLA. Facial expressions are presenting mixed findings: on one side, facial expressions in terms of happiness are always much higher for TESLA, also for Task 4. On the other side, confusion, one of the three new affective attitudes released by FaceReader 8.1, is showing significantly higher values for TESLA in comparison to FORD, detecting the negative impact raised by the seek for the “Assistance” label and the mental efforts to find it. It may be possible to explain the gap between these two outputs from automatic facial expressions analysis because happiness is a more general emotional reaction in comparison to confusion (Rozin and Cohen, 2003; Grafsgaard et al., 2011): happiness enrols a greater number of AUs and lasts for a shorter time in comparison to confusion, an affective state that shows up for 2 up to 5 s. These findings can also be explained through the strong customer-brand relationship that follows under the concept of brand love (Huber et al., 2016) that is “*the degree of passionate, emotional attachment a consumer has for a particular trade name”* (Carroll and Ahuvia, 2006). Trivedi and Sama (2020) categorised several antecedents of brand love, such as brand trust, CX, psychological attachment, and hedonic value of the brand, identifying how brand love is a strong indicator of a customer's affective response to the brand during the CX (Roy et al., 2013; Trivedi, 2019; Trivedi and Sama, 2020). Therefore, a possible interpretation of our results can rely on the moderation effects of brand love for TESLA, as shown by the ranking provided by Interbrand (https://www.rankingthebrands.com) where TESLA has been able to gain 59 positions in 2020 in comparison to FORD, whose position raised only 20 points. Overall, the design of the two websites seems to raise different emotional impacts: the TESLA website takes advantage of much more pictures and visual elements, as well as of colours and “3D virtual tours” that may represent one of the key elements, enabling a general greater emotional impact. For instance, considering now Task 2, subjects were instructed to look for the Tesla “Model X.” We choose, by purpose, this model, as the landing page of this model, once loaded by the internet browser, showed in the upper part of the page a “3D virtual tour” of the car from the front to the rear, with the peculiar doors opening like two “wings” of a seagull: the “3D virtual tour,” lasting 5 or 6 s and automatically starting once the webpage was opened, raised quite a big effect in terms of emotional reactions (see graph in picture 3 or the Results section, where the level of happiness is much higher in comparison to FORD, especially in the beginning part of the task, when actually, the participants were exposed to the “3D virtual tour” described). For the FORD website, where the model was asked to look for the “New Explorer,” this car model was presented by means of a landing page with classic pictures, videos (that could start only after clicking; thus, they could be considered as additional pictures with the “play icon” in the middle, as none of the participants decided to start a video) and longer text sections in comparison to the TESLA landing page. All emotional effects from these distinct elements and layouts are detected by the different levels of happiness showing up on the faces of the participants.

Aside from the specific web contents and “information architecture” styles and designs, the aim of the present research project was to show how emotional impact played by websites can be assessed by neuromarketing techniques such as automatic facial emotion detection, coupled with reaction time methods, which no previous research tried to investigate. With this work, it is possible to show how these techniques can be efficiently applied to website evaluation and widening insights to understand and assess UX.

## Conclusion

In our study, the data collected by means of automatic facial emotional expressions during website exploration and implicit association techniques applied before and after web navigation evidenced how different design solutions to shape UX. Moreover, it shows how the integration of neuromarketing techniques with traditional ones may enhance the understanding and evaluation of UX. These findings may have implications for developing new protocols for the user and usability testing.

## Data Availability Statement

The raw data supporting the conclusions of this article will be made available by the authors, with the permission of the companies (SR LABS and NEUROHM) that contributed to the research project realisation.

## Ethics Statement

Ethical review and approval was not required for the study on human participants in accordance with the local legislation and institutional requirements. The patients/participants provided their written informed consent to participate in this study.

## Author Contributions

All authors listed have made a substantial, direct and intellectual contribution to the work, and approved it for publication.

## Conflict of Interest

The authors declare that the research was conducted in the absence of any commercial or financial relationships that could be construed as a potential conflict of interest.

## Publisher's Note

All claims expressed in this article are solely those of the authors and do not necessarily represent those of their affiliated organizations, or those of the publisher, the editors and the reviewers. Any product that may be evaluated in this article, or claim that may be made by its manufacturer, is not guaranteed or endorsed by the publisher.

## Footnotes

^1^The “New Explorer” sold on the Ford Italy site is equivalent to the “2021 Explorer” sold on the U.S. site.

^2^The Ford “Mustang Bullitt” is no longer in production as of January 31, 2021 (Foote, 2021).
